# *COOLAIR* and PRC2 function in parallel to silence *FLC* during vernalization

**DOI:** 10.1073/pnas.2311474121

**Published:** 2024-01-18

**Authors:** Mathias Nielsen, Govind Menon, Yusheng Zhao, Eduardo Mateo-Bonmati, Philip Wolff, Shaoli Zhou, Martin Howard, Caroline Dean

**Affiliations:** ^a^Department of Cell and Developmental Biology, John Innes Centre, Norwich NR4 7UH, United Kingdom; ^b^Computational and Systems Biology, John Innes Centre, Norwich NR4 7UH, United Kingdom

**Keywords:** antisense transcription, Polycomb Repressive Complex 2, *FLC*, Arabidopsis

## Abstract

Noncoding transcription induces chromatin changes that can mediate environmental responsiveness, but the causes and consequences of these mechanisms are still unclear. Here, we investigate how antisense transcription (termed *COOLAIR*) interfaces with Polycomb Repressive Complex 2 (PRC2) silencing during winter-induced epigenetic regulation of Arabidopsis *FLOWERING LOCUS C* (*FLC*). We use genetic and chromatin analyses on lines ineffective or hyperactive for the antisense pathway in combination with computational modeling to define the mechanisms underlying *FLC* repression. Our results show that *FLC* is silenced through pathways that function with different dynamics: a *COOLAIR* transcription-mediated pathway capable of fast response and in parallel a slow PRC2 switching mechanism that maintains each allele in an epigenetically silenced state. Components of both the *COOLAIR* and PRC2 pathways are regulated by a common transcriptional regulator (NTL8), which accumulates by reduced dilution due to slow growth at low temperature. The parallel activities of the regulatory steps, and their control by temperature-dependent growth dynamics, create a flexible system for registering widely fluctuating natural temperature conditions that change year on year, and yet ensure robust epigenetic silencing of *FLC.*

## Significance

The role of noncoding transcription in chromatin regulation is still controversial, extending to the role of transcription of antisense transcripts called *COOLAIR* in the Polycomb-mediated epigenetic silencing of Arabidopsis *FLC* (*FLOWERING LOCUS C*), a key step during vernalization. Here, we show that *COOLAIR* transcription and PRC2 (Polycomb Repressive Complex 2) silence *FLC* in parallel pathways: an antisense-mediated transcriptional repression capable of fast response and a slow PRC2 epigenetic silencing, both of which are affected by growth dynamics and temperature fluctuations. These features explain the varied importance of *COOLAIR* transcription in cold-induced *FLC* epigenetic silencing seen in various studies using different conditions. The parallel repressive inputs and extensive feedbacks make the mechanism counterintuitive but provide great flexibility to the plant.

Noncoding transcription has emerged as an important mechanism in environmentally responsive gene regulation. In some cases, noncoding transcription induces chromatin changes that are lost if the environmental signal is removed (1, 2). In other cases, chromatin changes, particularly those involving the Polycomb mark H3K27me3, are epigenetically maintained providing a memory of the inductive signal. One well-characterized example of the latter is the winter-induced epigenetic silencing of the Arabidopsis floral repressor gene, *FLC* (*FLOWERING LOCUS C*) (3, 4). This underpins the vernalization process, the acceleration of flowering by winter exposure. The process includes early induction of a series of antisense transcripts, called *COOLAIR* (5); a slow epigenetic switch from an active chromatin environment (marked by H3K36me3) to a silenced chromatin state (marked by H3K27me3) at an internal three nucleosome region (6); and spreading of the H3K27me3 Polycomb silencing over the whole locus (7, 8). The switching mechanism involves canonical Polycomb Repressive Complex 2 (PRC2) and Arabidopsis PRC2 accessory proteins VIN3 and VRN5. VIN3 is slowly induced by cold exposure (9), interacts with PRC2 at the nucleation region downstream of the *FLC* transcription start site (TSS), and has a functionally important head-to-tail polymerization domain (10).

The timing of early antisense transcription and later VIN3 expression led to the view that antisense transcription was a prerequisite for PRC2 silencing. Consistent with this, single-molecule FISH experiments revealed that *COOLAIR* expression was mutually exclusive with *FLC* sense transcription at each allele (11). This sequence of events was initially tested through T-DNA insertions into the *COOLAIR* promoter. These had little effect on long-term vernalization (12). Similarly, *FLC* silencing was unaffected in studies using a CRISPR deletion of the *COOLAIR* promoter or mutation of *CBF* factors, known to facilitate cold induction of *COOLAIR* (13). However, replacement of *COOLAIR* 5′ sequences (TEX1 line) attenuated *FLC* transcriptional silencing and disrupted the coordinated changes in H3K36me3 and H3K27me3 occurring at the *FLC* nucleation region (14).

*COOLAIR* had much stronger effects in experiments analyzing *FLC* silencing in natural field conditions. *COOLAIR* expression was strongly induced on the first freezing night of autumn (15, 16), a result recreated in controlled environment cabinets (15). In these experiments, one freezing night was sufficient to induce *COOLAIR,* but several freezing nights were required to silence *FLC,* with silencing attenuated by disruption of antisense transcription. These data are reminiscent of many *Saccharomyces cerevisiae* loci, where noncoding transcription plays an important role in environmental responsiveness (1, 17, 18). However, extensive feedback mechanisms between chromatin, transcription and cotranscriptional processes make functions of noncoding transcription difficult to elucidate. In particular, buffering between transcription and RNA stability leads to changed transcriptional dynamics with no change in steady state RNA (2).

To clarify the regulatory mechanism at Arabidopsis *FLC,* we have undertaken a series of genetic, molecular, and computational analyses to investigate the role of *COOLAIR* in cold-induced *FLC* silencing. Here, we show that *FLC* is silenced through parallel pathways. *COOLAIR* transcription can limit sense transcription, and this is associated with reduction in levels of the active histone mark H3K36me3; this mechanism involves disruption of a 5′-3′ *FLC* gene loop (19). In parallel, PRC2 silencing switches each allele from an epigenetically ON to an OFF state; this involves nucleation of H3K27me3 and subsequent spreading over the locus during subsequent growth, associated with further reduction in H3K36me3 (6). The nucleated and spread states differentially influence *FLC* transcription, which is still modulated by *COOLAIR* transcription. While *FLC* silencing by the PRC2 pathway operates on a slow timescale, the rapid induction capability of *COOLAIR* transcription, as seen in freezing conditions (15), enables this pathway in these conditions to silence *FLC* transcription on fast timescales. Components of both pathways are also regulated by their common transcriptional regulator NTL8 (20), which accumulates based on reduced dilution dependent on growth dynamics in the different cold phases. We integrate these parallel regulatory activities into a mathematical model that predicts *FLC* chromatin dynamics and transcription in different conditions. We argue that parallel activities converging onto a common target provides great flexibility in gene regulation, providing responsiveness to a wide variety of conditions. There are extensive similarities between how antisense transcription modulates *FLC* and how it alters sense transcription dynamics in yeast (2).

## Results

### *COOLAIR* Rather than PRC2 Nucleation is the Major Contributor to *FLC* Repression in *ntl8-D3*.

**Fig. 1.**
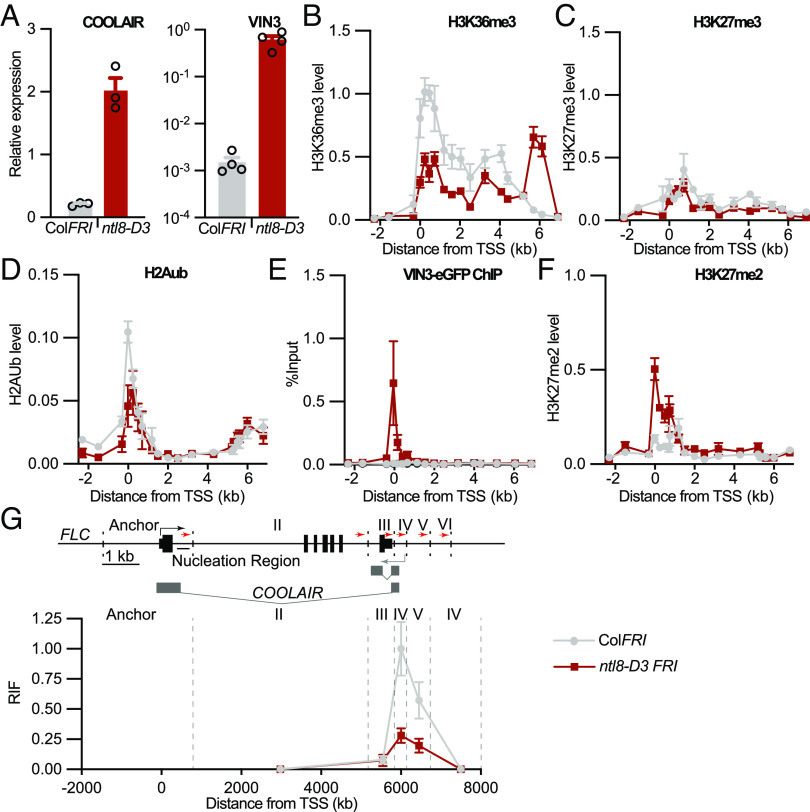
*ntl8-D3* mimics cold exposure, except for the accumulation of H3K27me3 and H2Aub. (*A*) Expression of total *COOLAIR* and *VIN3* in *ntl8-D3 FRI* and Col*FRI* in nonvernalized conditions (NV). Data are presented as the mean ± SEM. Each open circle represents a biological replicate. (*B*–*D*) Enrichment of (*B*) H3K36me3, (*C*) H3K27me3, and (*D*) H2Aub across *FLC* measured by ChIP in wild-type Col*FRI* and *ntl8-D3* at NV conditions. H3K36me3 data are shown relative to H3 and actin. H3K27me3 data are shown relative to H3 and STM. H2Aub data are shown relative to H3. Error bars represent SEM (n ≥ 3 biological replicates). (*E*) VIN3-eGFP ChIP-qPCR enrichment at *FLC* at NV. Data are shown as the percentage input. Nontransgenic Col*FRI* plants were used as a negative control sample. Error bars represent SEM (n = 3 biological replicates). (*F*) Enrichment of H3K27me2 across *FLC* measured by ChIP in wild-type Col*FRI* and *ntl8-D3* at NV conditions. Data are expressed relative to H3. Error bars represent SEM (n = 3 biological replicates). (*G*) Quantitative 3C-qPCR over the *FLC* locus in 10-d-old Col*FRI* and *ntl8-D3 FRI* seedlings. A schematic representation of the *FLC* locus is shown above. BamHI and BglII restriction sites are indicated with dotted lines, and the respective regions are numbered with Roman numerals. Red arrows indicate the location of the primers used for 3C-qPCR. The region around the *FLC* TSS was used as the anchor region in the 3C analysis. The data below show the relative interaction frequencies (RIF).

Two independent genetic screens in different genotypes had identified dominant mutations that revealed NTL8 regulates *VIN3* and *COOLAIR* (15, 20). Ectopic *COOLAIR* expression leads to very low *FLC* levels in warm grown plants (15). We therefore confirmed that *VIN3* and *COOLAIR* are both misregulated in the dominant mutant *ntl8-D3* (Fig. 1*A*), and then used it to genetically activate both pathways simultaneously, independently of cold. *FLC* transcriptional output, histone modifications and chromatin topology were analyzed. Paralleling cold effects on wild-type plants the ectopic *COOLAIR* expression in *ntl8-D3* resulted in a clear decrease in H3K36me3, as compared to Col*FRI,* at the *FLC* TSS and over the gene body (Fig. 1*B*). The high *COOLAIR* transcription in *ntl8-D3* led to accumulation of H3K36me3 at the *COOLAIR* promoter (Fig. 1*B*), matching the cold-induced transient increase of H3K36me3 in Col*FRI* at the same position. The decrease in H3K36me3 was not accompanied by an increase in H3K27me3 observed during vernalization (Fig. 1*C*). Likewise, H2Aub, another histone modification that accumulates at *FLC* during early vernalization did not accumulate ectopically in *ntl8-D3* (Fig. 1*D*). The lack of accumulation of H3K27me3 and H2Aub in *ntl8-D3* compared to Col*FRI* in the absence of cold supports the view that VIN3 expression itself is not sufficient to cause Polycomb mediated silencing of *FLC*. These data indicate that antisense-mediated suppression rather than VIN3-mediated nucleation of H3K27me3 is the major factor causing *FLC* repression in *ntl8-D3*. Repression of sense *FLC* transcription in *ntl8-D3* is almost completely suppressed when *COOLAIR* transcription is blocked, giving further support to this conclusion (15).

### Ectopically Expressed VIN3 Localizes to *FLC* but Fails to Induce H3K27me3 Nucleation.

To understand what prevents the accumulation of H3K27me3 in *ntl8-D3* despite ectopic VIN3 expression, we tested whether other epigenetic factors are misexpressed in *ntl8-D3.* Only one of the tested genes changed slightly in expression (*SI Appendix*, Fig. S1). We then analyzed association of VIN3 at the nucleation region in *ntl8-D3*. Despite the lack of H3K27me3 accumulation in *ntl8-D3*, we found VIN3-eGFP accumulated at the *FLC* nucleation region in warm conditions, mimicking the accumulation during vernalization (Fig. 1*E*). Thus, VIN3 accumulation at the nucleation region does not result in stable nucleation of H3K27me3. To distinguish VIN3 intrinsic binding to the *FLC* nucleation region, independently of *COOLAIR* transcriptional induction, we expressed VIN3-eGFP under the promoter of VRN5 (*SI Appendix*, Fig. S2*A*). This resulted in expression levels in nonvernalized plants that paralleled VIN3 induction after 6 wk cold (6WT0) (*SI Appendix*, Fig. S2*B*). In this line VIN3-eGFP was enriched at the *FLC* locus in NV conditions (*SI Appendix*, Fig. S2*C*), showing VIN3 can remain associated with the nucleation region even when *FLC* is strongly expressed. We found that VIN3 association in *ntl8-D3* led to H3K27me2 enrichment despite no accumulation of H3K27me3 (Fig. 1*F*). Thus, cold-induced features, possibly influencing residence time, are required to enable VIN3 functionality to deliver H3K27me3 to the nucleation region.

### Ectopic Induction of *COOLAIR* Correlates with Chromatin Topology Changes.

A cold-induced feature at *FLC* is disruption of a gene loop conformation that links the TSS and the transcription termination site (19). In *ntl8-D3,* we found that the gene loop was ectopically disrupted, mimicking vernalization (Fig. 1*G*). This suggests that gene loop disruption is linked with antisense-mediated reduction in *FLC* transcription. We also found that the TEX2.0 transgene, where a *nos* terminator promotes early *COOLAIR* termination, reduces gene loop formation (*SI Appendix*, Fig. S3), consistent with earlier reports using a similar, but not identical transgene (21). This result suggests a role for the activity of the antisense promoter/TSS, rather than antisense transcription per se, as being important for gene loop disruption.

### Disrupting *COOLAIR* Transcription Perturbs H3K27me3 Dynamics Before and during Cold, but Not Postcold H3K27me3 Levels.

We further investigated the fact that ectopic expression of antisense transcription is enough to cause lower H3K36me3 around the *FLC* sense TSS and in the gene body, even in the absence of cold. Antisense transcription could lower H3K36me3 levels, either through direct removal mediated by antisense transcription or indirectly by limiting sense transcription, thus preventing the cotranscriptional addition of H3K36 methylation. To dissect the interplay of H3K36me3 and H3K27me3, we studied the dynamic changes in these modifications using a vernalization time course.

**Fig. 2.**
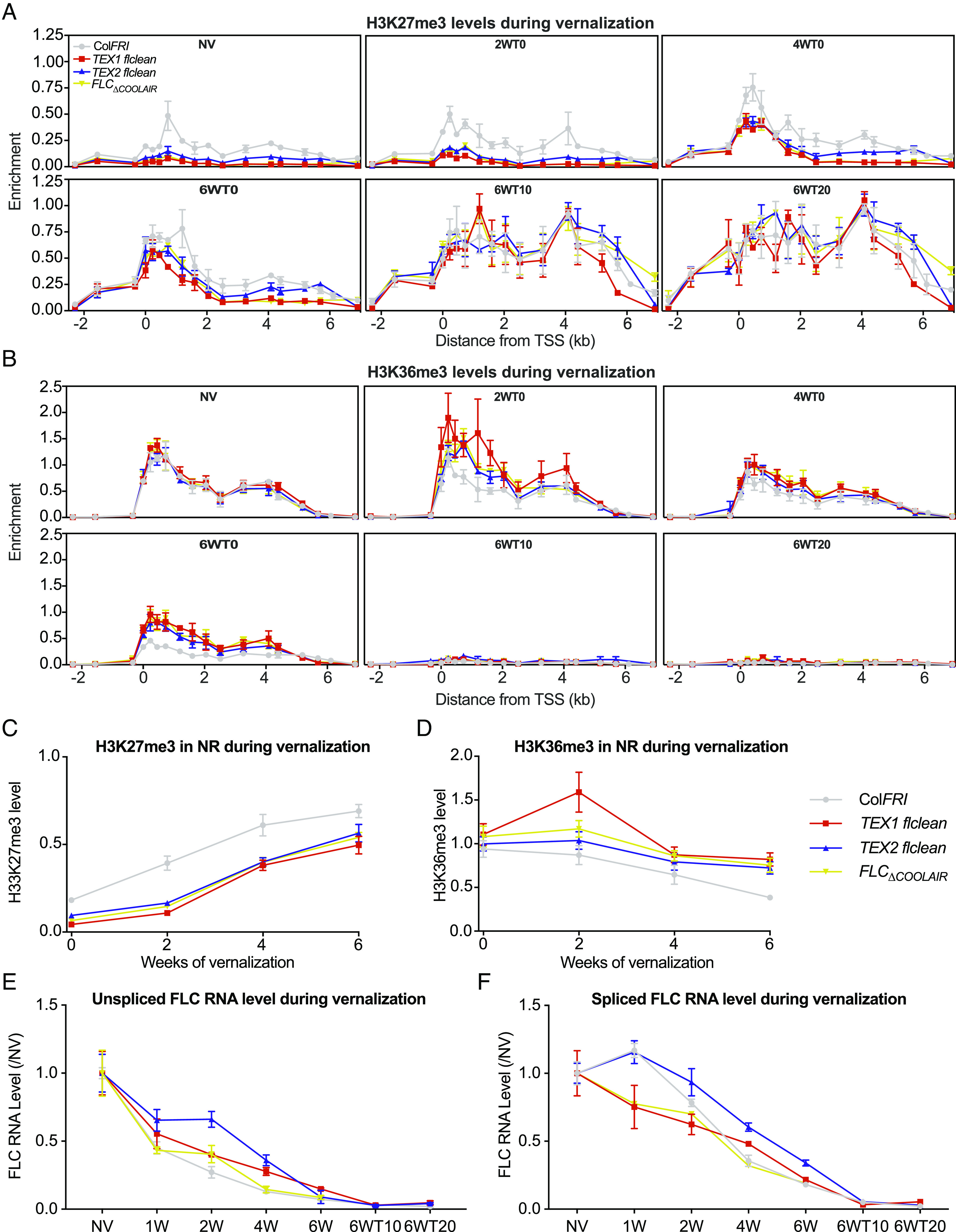
Cold-induced chromatin and RNA dynamics in *COOLAIR* defective lines. (*A* and *B*) Enrichment of H3K27me3 (*A*) and H3K36me3 (*B*) across *FLC* measured by ChIP in wild-type Col*FRI* and the three defective *COOLAIR* lines, *TEX1, TEX2,* and *FLC*_Δ*COOLAIR*_, before, during, and after vernalization. Data are expressed relative to H3 and *STM*. Error bars represent SEM (n = 3 biological replicates). (*C* and *D*) Average levels of H3K27me3 (*C*) and H3K36me3 (*D*) in the nucleation region during vernalization. The averages were calculated by averaging the ChIP enrichment over three primers in the *FLC* nucleation region during vernalization in Col*FRI* and each of the defective *COOLAIR* lines. (*E* and *F*) *FLC* expression during a vernalization time course in Col*FRI* and the three defective *COOLAIR* lines, unspliced (*E*) and spliced RNA (*F*), was measured and is shown relative to *UBC* and NV levels. Error bars represent SEM (n = 3 biological replicates).

Our previous analyses of TEX transgenes were in an *flc-2* background, where part of the endogenous *FLC* genomic sequence remains (4). This limited the regions where the chromatin modifications on the transgene could be studied (14). To overcome this limitation, we generated a FRI + *FLC* null (*flclean*) where the entire *FLC* genomic sequence had been deleted using CRISPR (*SI Appendix*, Fig. S4) and introduced the previously described TEX1.0 (replacement of the *COOLAIR* promoter) and TEX2.0 (insertion of a *nos* terminator to truncate *COOLAIR* transcription) transgenes. We also included a *FRI FLC*_Δ*COOLAIR*_ CRISPR line, which deletes the *COOLAIR* promoter at the endogenous locus (22). Using these multiple defective *COOLAIR* lines and respective controls, we undertook a detailed time course of histone modifications during vernalization, including multiple time points postcold (Fig. 2 *A* and *B*).

The rate of accumulation of H3K27me3 during cold exposure was not reduced in the *COOLAIR* defective genotypes compared to the wild type, and at some timepoints was even accelerated (Fig. 2*C* and *SI Appendix*, Fig. S5 *A* and *B*), consistent with our previous data (14). By 6WT0, wild-type and *COOLAIR* defective genotypes show similar H3K27me3 levels in the nucleation region (Fig. 2*A* and *SI Appendix*, Fig. S5*C*). However, there were clear differences in the starting levels of H3K27me3, being significantly lower in the nucleation region in all defective *COOLAIR* genotypes (Fig. 2*A* and *SI Appendix*, Fig. S5*D*). Consistent with the differences in starting H3K27me3 levels, and supporting a role for antisense transcription in establishment of the initial *FLC* chromatin state (23), the defective *COOLAIR* genotypes showed a consistent trend of higher *FLC* RNA before cold exposure (*SI Appendix*, Fig. S5*E*), although the differences were small. The similar trend in TEX1, TEX2 and *FLC*_ΔCOOLAIR_ argues against this being a specific TEX transgene effect. We interpret the H3K27me3 level in Col*FRI* before cold as representing a fraction of *FLC* alleles that have switched to a stable Polycomb silenced state. Thus, higher H3K27me3 levels in Col*FRI* compared to the *COOLAIR* defective genotypes may reflect the *COOLAIR* role in developmentally regulated PRC2 silencing of *FLC* (24, 25). After cold there was no significant difference in H3K27me3 levels in the nucleation region between Col*FRI* and any of the *COOLAIR* defective genotypes (Fig. 2*A* and *SI Appendix*, Fig. S5*C*). Spreading of H3K27me3 was also unaffected in the *COOLAIR* defective genotypes, as seen from the similar levels in the gene body at 6WT10 and 6WT20 (Fig. 2*A*). Overall, we find that H3K27me3 dynamics before and during cold are perturbed by *COOLAIR*, but that postcold H3K27me3 levels are not.

### Disrupting *COOLAIR* Transcription Attenuates H3K36me3 Removal during Vernalization.

H3K36me3 levels were similar in all genotypes before vernalization (Fig. 2*B* and *SI Appendix*, Fig. S5*F*) but decreased at different rates during cold exposure (Fig. 2*D*). This contrasts with the clear NV differences in H3K27me3 levels. However, this is consistent with the NV H3K27me3 levels coming from a small fraction of silenced alleles, while most alleles are transcriptionally active and contribute to the observed H3K36me3 levels, a scenario that generates bigger fold changes in H3K27me3 than in H3K36me3, as we observe (*SI Appendix*, Fig. S5*G*). H3K36me3 levels reduced more slowly in all defective *COOLAIR* genotypes at 6WT0 (Fig. 2*B* and *SI Appendix*, Fig. S5*G*), but after 2 wk cold H3K36me3 levels increased in the gene body compared to NV (Fig. 2*B*). There were no differences in H3K36me3 levels between *COOLAIR* defective genotypes and wild-type Col*FRI* after transfer back to warm (Fig. 2*B*). In *ntl8-D3*, where *VIN3* and *COOLAIR* are both overexpressed, faster reduction of H3K36me3 in the cold was observed, while H3K27me3 was less affected (*SI Appendix*, Figs. S6 *A*–*D* and S9 *B* and *C*). Together our results demonstrate that the Polycomb pathway is effective enough to completely silence the *FLC* locus, despite either an ineffective or hyperactive antisense pathway. The *COOLAIR*-mediated pathway mediates not only the removal of H3K36me3 but also H3K4me1 through the activity of the demethylase complex FLD-LD-SDG26 (23). H3K4me1, like H3K36me3, has been shown to be added cotranscriptionally in plants (26). Consistently, we found that in the *COOLAIR* defective lines, H3K4me1 reduction during vernalization was attenuated (*SI Appendix*, Fig. S7) showing the same trend as H3K36me3, including the increase at 2WT0. Overall, we find that *COOLAIR* defective genotypes have reduced rates of H3K36me3 removal, but after cold, any differences in H3K36me3 levels disappear. The relative changes of the unspliced *FLC* RNA levels did not match the corresponding H3K36me3 levels in the *COOLAIR* defective genotypes and effects on spliced *FLC* levels were different to unspliced (Fig. 2 *E* and *F*). This suggests a similar interconnected mechanism linking chromatin modification to transcript stability as found in yeast, with unspliced and spliced transcripts affected in different ways (2).

### H3K27me3 Accumulation Is Not Necessary for *COOLAIR-*Mediated Transcriptional Downregulation.

**Fig. 3.**
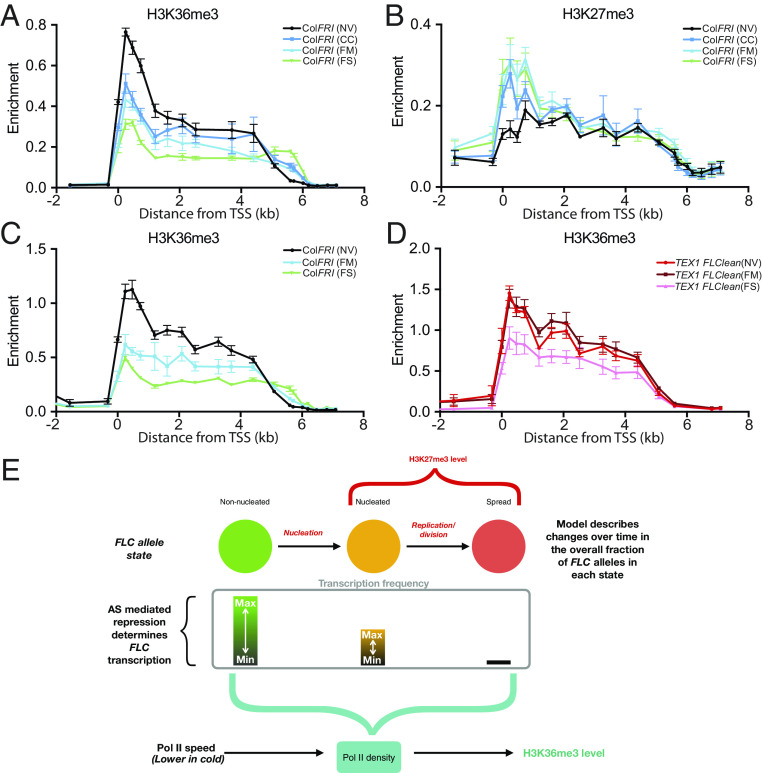
Fluctuating cold and mathematical modeling of the role of *COOLAIR* in histone modification dynamics. (*A* and *B*) Changes in H3K36me3 (*A*) and H3K27me3 (*B*) at *FLC* after 2 wk of CC, FM, or FS conditions, measured by ChIP-qPCR. Data are expressed relative to H3. Error bars represent SEM (n = 3 biological replicates). (*C* and *D*) Comparing changes in H3K36me3 at *FLC* between Col*FR*I (*C*) and *TEX1 flclean* (*D*) after 2 wk of FM or FS conditions, measured by ChIP-qPCR. Data are expressed relative to H3 and *STM*. Error bars represent SEM (n = 3 biological replicates). (*E*) Schematic of the mathematical model showing core components: PRC2-mediated silenced states (non-nucleated, nucleated, and spread) at individual *FLC* alleles, antisense transcription–mediated repression of *FLC* transcription, and the contribution of these components to the average population-level H3K36me3 coverage at the *FLC* locus.

A mutation in the core PRC2 component Su(z)12 (VRN2) only partially disrupted *FLC* repression (*SI Appendix*, Fig. S8*A*) (6), while H3K36me3 fold reduction at *FLC* during the cold was hardly changed (*SI Appendix*, Fig. S8*B*), despite accumulation of H3K27me3 being abolished (6). Thus, H3K36me3 reduction and *FLC* RNA downregulation do not rely on H3K27me3 nucleation. Analysis of a *vrn5-TEX1.0* combination, defective in H3K27me3 accumulation and *COOLAIR*, had shown that the H3K36me3 reduction seen in an H3K27me3 nucleation mutant is mediated by *COOLAIR* (14). To examine this aspect further, we analyzed changes in the two modifications, H3K36me3 and H3K27me3, in fluctuating cold conditions, where we see the clearest indication of *COOLAIR* transcription regulating *FLC* expression (15). Under these conditions, *COOLAIR* was highly up-regulated, causing significant downregulation of *FLC* sense transcript (15). While we have previously shown that full-length *COOLAIR* transcription is essential for the *FLC* downregulation in these conditions (15), a role for H3K27me3 nucleation had not been investigated. Here, we analyzed H3K36me3 and H3K27me3 levels in Col*FRI* at 2WT0, under three different cold conditions (as in ref. 15), constant 5 °C (CC, Constant Cold), mild 3 to 9 °C (FM, Fluctuating Mild), and strong fluctuating conditions −1 to 12 °C (FS, Fluctuating Strong). Zhao et al. (15) showed that *COOLAIR* upregulation and *FLC* downregulation were greatest in the FS condition. Therefore, we would expect H3K36me3 to show the largest changes in this condition, and indeed this is seen in our data (Fig. 3*A* and *SI Appendix*, Fig. S9*E*). Both mild fluctuating and CC result in smaller changes (Fig. 3*A* and *SI Appendix*, Fig. S9*E*). In contrast, H3K27me3 accumulation showed little difference between the different conditions (Fig. 3*B* and *SI Appendix*, Fig. S9*D*), indicating that H3K27me3 is not the major contributor to the enhanced downregulation under strongly fluctuating conditions. The lack of difference in H3K27me3 accumulation, despite the relatively large change in antisense expression, further highlights the parallel and almost independent nature of these *FLC* repression pathways. To further confirm that *COOLAIR* transcription is necessary for the changes in H3K36me3 under fluctuating conditions, we subjected the TEX1.0 defective *COOLAIR* line to these conditions. As expected, the reduction in H3K36me3 was also attenuated relative to Col*FRI* (Fig. 3 *C* and *D*). This is consistent with the lack of *FLC* sense transcriptional shutdown under FS conditions in a *COOLAIR* deletion line as recently described (27). Interestingly, for *COOLAIR* defective genotypes, the slight increase in H3K36me3 at 2WT0 observed in CC was also recapitulated in the FM conditions. The reduction under FS conditions was significantly attenuated in TEX1.0 (*SI Appendix*, Fig. S9*F*), and an analysis to test whether this is also the case in the *COOLAIR* deletion line is ongoing. Overall, we find that *COOLAIR*-mediated transcriptional repression does not strongly depend on H3K27me3 nucleation, supporting our earlier results in *ntl8-D3*. The contrasting dynamics of H3K36me3 and H3K27me3 under 2 wk of fluctuating conditions further highlight the fast-response capability of the antisense-mediated repression. In Col*FRI*, the changes in nucleation region H3K36me3 after only 2 wk of FS conditions (Fig. 3 *A* and *C*) are comparable to H3K36me3 changes after 6 wk in CC (Fig. 2*B* and *SI Appendix*, Fig. S5*G*). The fast response capability of *FLC* antisense transcription to temperature changes is also supported by field data for A. *halleri FLC* (28). They showed that H3K4me3 associated with *COOLAIR* transcription at the 3′ end of the *FLC* locus responds to temperature changes on a much faster timescale compared to the 5′ end (associated with *FLC* sense transcription), which responds mainly on a slow timescale.

### Mathematical Modeling of *FLC* Regulation Reconciles the Different Effects of Antisense Transcription on Chromatin State.

The dynamics of the two repression pathways are difficult to dissect quantitatively purely through molecular experiments. We therefore turned to mathematical modeling to see how the observed behavior in *COOLAIR* defective mutants could be reconciled with our existing understanding of *FLC* repression in the cold. We have previously developed and experimentally validated a mathematical modeling framework describing dynamically changing fractions of active/silenced *FLC* alleles and their associated histone modifications (29, 30). Here, we built on this framework to develop an augmented model, incorporating an antisense-mediated silencing component. A schematic of the model developed here is shown in Fig. 3*E* (details in *SI Appendix*—the MATLAB code used to simulate the ODE model is available at https://doi.org/10.5281/zenodo.10257997). The model was built based on our main conclusion from the above data: namely that two pathways work in parallel to silence *FLC*, antisense transcription and PRC2 nucleation. We then interrogated the model to see whether it was capable of quantitatively reproducing histone modification dynamics in Col*FRI* and the various mutants.

The effect of the antisense-mediated pathway on sense transcription was modeled implicitly as a cold-dependent graded modulator of sense initiation/transition to productive elongation. This is consistent with high antisense transcription in *ntl8-D3* causing low levels of *FLC* transcription, independently of H3K27me3 nucleation. This is also consistent with previous data showing that sense and antisense transcription at *FLC* are anticorrelated in Col*FRI*, both in warm (31) and in cold conditions (11). This may be through a mutual exclusivity model for the *FLC* locus, similar to that reported for the *CBF1-SVALKA* locus, where full-length sense transcription is inhibited by antisense transcription (32). Another key aspect of the model is the cotranscriptional delivery of the H3K36me3 modifications. Changes in Pol II elongation behavior can affect the H3K36me3 profile across mammalian genes (33), with slower Pol II speed allowing a larger window of opportunity for adding H3K36me3 at any given location. Any changes in transcription at *FLC* may be expected to produce corresponding changes in H3K36me3. However, to explain the *increase* in gene body H3K36me3 observed in the defective *COOLAIR* lines, specifically at 2WT0 compared to NV, despite the lack of any increase in transcriptional output over that time period (Fig. 2*E*), the model includes a cold-induced reduction in Pol II speed in this region, resulting in a longer dwell time. The SDG8 H3K36 methyltransferase, which we have shown cotranscriptionally associates with RNA PolII (34), is likely mediating these H3K36me3 changes. The model also allows for H3K36me3 removal on a timescale consistent with the experimentally observed lifetime of H3K36 methylation in other systems (35), so that its levels at the nucleation region would decay quickly in the absence of sense transcription. The model also describes dynamic changes in *FLC* mRNA levels as modeled in ref. 29. However, due to the highly variable behavior of spliced *FLC* RNA observed in the different *COOLAIR* defective mutants (Fig. 2*F*), which potentially reflects changes in RNA stability, we do not try to capture these levels using the model.

**Fig. 4.**
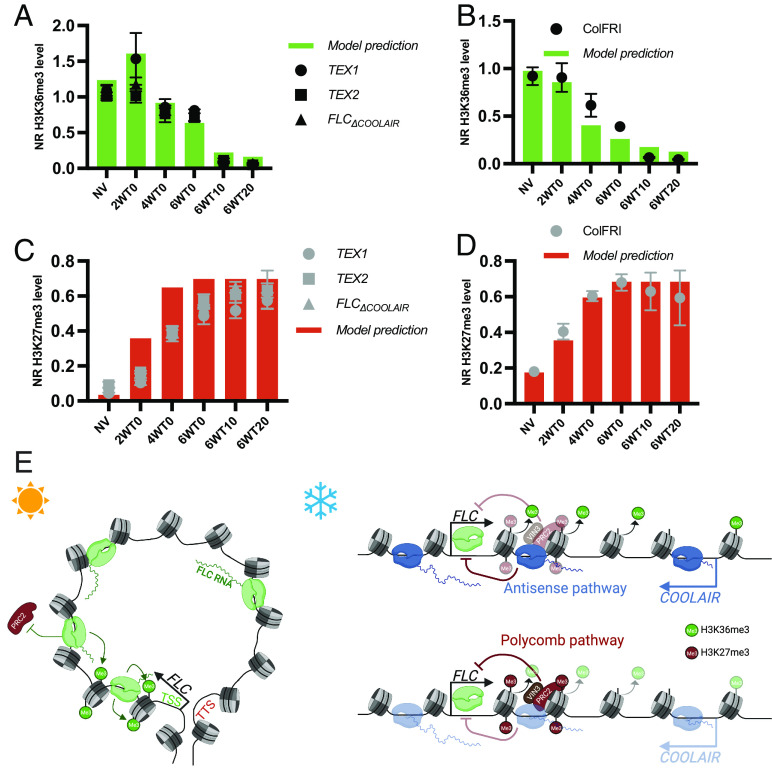
Model predictions of the impact of vernalization mutants on histone dynamics and schematic representation of parallel pathways that repress *FLC* expression. (*A*–*D*) Mathematical model predicted levels of H3K36me3 (*A* and *B*) and H3K27me3 (*C* and *D*) over a CC time course in a defective *COOLAIR* mutant (*A* and *C*) and the wild type, Col*FRI* (*B* and *D*). The predictions are compared to the ChIP-qPCR time course data for the different genotypes presented in Fig. 2 (*A* and *B*). (*E*) Model for the parallel pathways that repress *FLC*. In the warm *FLC* forms a gene-loop conformation, which mediates a high expression state of *FLC*. The high expression state is marked by high levels of H3K36me3 around the *FLC* TSS. After cold exposure, the repressive pathways are activated: 1) the antisense-mediated pathway leading to disruption of the gene loop and removal of H3K36me3 from the TSS of *FLC* and 2) the Polycomb pathway leading to deposition of H3K27me3 and repression of *FLC* transcription. The two pathways work in parallel rather than through a linear sequence of causation to give the final *FLC* expression outcome during vernalization.

We also incorporate the PRC2 pathway and how it silences *FLC* through H3K27me3 accumulation during vernalization. In cells which can have active or nonactive cell cycles, we consider that *FLC* alleles can be in three different states; non-nucleated (without H3K27me3 nucleation), nucleated, and spread, with the latter only attained in active cycling cells (29). To generate reasonable fits to our data, particularly the higher levels of H3K36me3 observed during cold in *COOLAIR* defective mutants, we found that an extension to our previous models was needed, where we allow for different levels of *FLC* transcription in the three states: highest in non-nucleated, much lower in nucleated, and even lower in spread. Satisfactory fits also necessitated that the non-nucleated and nucleated states be capable of further downregulation by antisense transcription. Consistent with the possibility of some limited transcriptional activity in the nucleated state, we, therefore, allowed for potential coexistence of H3K27me3 and H3K36me3 on the same nucleosome. We then fitted the model to capture the qualitative changes in H3K36me3, H3K27me3, and transcription (sense and antisense) observed in the cold for Col*FRI* and the defective *COOLAIR* lines. We found that this model could capture all the qualitative features of the data observed in Col*FRI* and the defective *COOLAIR* lines (Fig. 4 *A*–*D*), including both the increase of H3K36me3 at 2WT0 and the subsequent significantly slower reduction in H3K36me3 in the latter (Fig. 4 *A* and *B*), as well as the reduction of H3K36me3 in the postcold seen in all the lines (Fig. 4 *A* and *B*).

We then tested whether the model could capture our previous datasets by simulating other mutants that affect *FLC* silencing in the cold [see previously published data (6)], including an H3K27me3 nucleation mutant (e.g., *vrn2, vin3*), a spreading mutant (e.g., *lhp1, clf)*. In all cases, the simulation outputs from the model are qualitatively consistent with data, including the postcold behavior of the two histone modifications (*SI Appendix*, Fig. S10 *A*–*D*). Interestingly, in addition to recapitulating the behavior captured by our previous models, the current model can capture the reduction of H3K36me3 in the postcold seen in Col*FRI*—a trend that could not be previously captured (Fig. 4*B*). This is because the current model allows for higher levels of transcription (and consequently higher H3K36me3) in a nucleated state relative to a spread state.

The model also incorporates a fast timescale response in the antisense mediated pathway, which can respond to temperature fluctuations (see *SI Appendix* for details). Briefly, this consists of a simple step increase in antisense mediated repression resulting from temperatures dropping below a threshold, which is incorporated into the slower timescale increase in antisense mediated repression. The fast timescale response allows the model to qualitatively capture the differences in H3K36me3 and *FLC* spliced RNA between the different cold conditions CC, FM, FS (*SI Appendix,* Fig. S11). The model predicts that H3K36me3 and *FLC* mRNA levels respond on a fast timescale, exhibiting oscillations in response to the daily repeated temperature profiles of FM and FS conditions. While the agreement between the model and experiments is overall good, the model did predict reduced H3K27me3 nucleation in FS conditions, which was not observed experimentally. This discrepancy may potentially arise from differences between the field conditions used to parameterize our model for H3K27me3 nucleation (30) and the FS experimental conditions used here. The model predicts that antisense transcription limits H3K36me3 through a graded, analog reduction in *FLC* transcription rather than by directly mediating H3K36me3 removal. The increased H3K36me3 at 2WT0 in *COOLAIR* defective lines, is predicted to arise from a combination of higher *FLC* sense transcription (since antisense mediated repression is disrupted) and cold-induced reduction in Pol II speed in the nucleation region. In a second slower response chromatin pathway involving PRC2, each allele progressively switches from a non-nucleated to H3K27me3 nucleated state during the cold and then to a spread H3K27me3 state during postcold growth. The model indicates that intermediate levels of *FLC* transcription in the nucleated state, which can be further down-regulated by antisense transcription, can explain how clear differences in H3K36me3 between defective *COOLAIR* lines and the wild type can emerge in the cold yet subsequently disappear during growth after transfer to warm conditions. In these conditions, all the nucleated *FLC* alleles convert to the H3K27me3 spread state due to an active cell cycle (6), regardless of H3K36me3 levels and any residual expression. Hence, in the context of vernalization, the *COOLAIR* repressive pathway is most important during rather than after cold.

## Discussion

Focused dissection of the mechanism underlying winter-induced *FLC* silencing has established a role for antisense transcription and PRC2 activity in registration of long-term exposure to noisy environmental signals (5, 15, 36, 37). However, the complexity of the mechanism, and its sensitivity to variable temperature and growth parameters, has led to different studies questioning the importance of the antisense transcription in cold-induced *FLC* silencing. Here, using a combination of experiments and mathematical modeling, we have elucidated the role of antisense transcription and PRC2 activity as parallel pathways, both leading to *FLC* silencing (Fig. 4*B*). The antisense-mediated pathway involves the *FLC* gene loop and represses *FLC* transcription (38). Two other lncRNAs have been described at *FLC*, *COLDWRAP* (39) and *COLDAIR* (40). We have detected cold-up-regulated *FLC* transcripts with upstream TSSs including *COLDWRAP* that influence *FLC* expression levels but not cold-induced transcriptional silencing (41); we have not found *COLDAIR* equivalents.

The multiple effects of the *COOLAIR*-mediated transcriptional pathway on H3K36me3 in the 5′ region of *FLC* required modeling to deconvolve fully. In the wild type, lower transcription leads to H3K36me3 reduction, but this is partly hidden in defective *COOLAIR* lines in the cold through a predicted increase in H3K36me3 from slower RNA PolII speed at the 5′ end of *FLC*. The slow PRC2 switch at each *FLC* allele from a non-nucleated to a H3K27me3 nucleated and then spread state is associated with decreasing frequencies of *FLC* transcription, consistent with previous findings of the relationship between H3K27me3 and *FLC* transcription (6). Both *COOLAIR*-mediated and PRC2 pathways are affected by the common transcriptional regulator NTL8, which accumulates slowly and variably dependent on reduced dilution by slower growth at low temperatures (20). Our observation that in *ntl8-D3* both *COOLAIR* and *VIN3* are ectopically expressed, yet the H3K27me2 modification but not H3K27me3 accumulates, implies a requirement for other cold-induced factors for vernalization (42, 43). These parallel repressive activities with multiple temperature inputs enables modulation of transcriptional silencing independently of robust epigenetic silencing. This gives the plants great flexibility to respond to autumnal conditions that vary in different geographical regions and from year to year yet ensure robust silencing. Indeed, variation in *FLC* transcriptional silencing has been shown to be an important adaptive determinant in *Arabidopsis thaliana* accessions (16). It seems likely that similar parallel mechanisms may be involved in other seasonal responses, e.g., seed and bud dormancy and germination, for similar reasons.

The chromatin changes in *COOLAIR* defective mutants are not directly reflected in steady-state unspliced and spliced *FLC* levels (Fig. 2 *E* and *F*), similar to the situation in yeast (2). Which RNA stability mechanisms are involved remain to be determined, but m6A methylation has been shown to influence *FLC* regulation (38, 44, 45) and is enriched in the *FLC* 3′ UTR. This disconnect between chromatin dynamics and steady-state RNA levels is likely to have contributed to the controversy over the role of noncoding RNA in chromatin regulation generally. In addition, the effective combination of parallel pathways hides effects of mutations after saturating induction, e.g., mutations in CBF-binding factors (13). Another debate has been over the use of transgenes to modulate *COOLAIR* expression (13), but the use here of *FLC*_Δ*COOLAIR*_ and *ntl8-D3* for under/overexpression of *COOLAIR* argues against this. However, future studies need to generate a fully antisense null genotype since all defective *COOLAIR* genotypes so far produced still contain cryptic antisense promoters, which become more active when the endogenous *COOLAIR* promoter is mutated/deleted (15). The difficulty of completely removing antisense transcription is also seen in other systems (46) and suggests transcription initiation from open chromatin regions rather than specific promoter elements. Such a line would not only help elucidate the role of *COOLAIR* in the cold-induced silencing of *FLC* but also in the starting *FLC* expression upon germination, a key determinant of natural variation underpinning adaptation (16).

The large number of plant chromatin regulators that interact with noncoding RNAs points to an important role of similar cotranscriptional mechanisms in environmental plasticity (47, 48). This is similarly true in yeast (2), where antisense expression has been associated with genes that are environmentally silenced (1). Future work will address the evolutionary parallels and conservation of a mechanism enabling rapid transcriptional changes and switches to epigenetic silencing in response to noisy environmental cues.

## Materials and Methods

Detailed descriptions of materials and methods are provided in *SI Appendix*. A brief summery is provide here.

### Plant Materials.

All mutant and transgenic lines were in the *FRI^sf2^* background. *ntl8-D3 FRI* was described previously (15). Generation of new mutant and transgenic lines is detailed in *SI Appendix*.

### Expression Analysis.

RNA analysis was performed as previously described (49). Total RNA was extracted and genomic DNA contamination removed using TURBO DNase (Invitrogen). cDNA was synthesized with SuperScript IV reverse transcriptase (Invitrogen). qPCR was performed using SYBR Green I Master (Roche) and analyzed on a LightCyler 480 machine (Roche).

### Chromatin Immunoprecipitation (ChIP).

ChIP was performed as described in ref. 49. ChIP was performed with antibodies: α-H3 (Abcam, ab1791), α-H3K36me3 (Abcam, ab9050), α-H3K27me3 (Abcam, ab192985), α-H3K27me2 (Upstate, 07-452), α-H2AK119ub (Cell Signaling Technology, #8240), α-H3K4me1 (Abcam, ab8895), and α-GFP (Abcam, ab290).

### Chromatin Conformation Capture.

Chromatin conformation capture was performed as described previously described (19) with minor modifications.

### Mathematical Modeling.

The models used in this study are constructed within a framework we have previously developed and experimentally validated (29, 30). In addition to what is captured by previous models, our current model also incorporates the dynamics of H3K36me3 and H3K27me3 at *FLC,* as observed in Col*FRI* and the *COOLAIR* defective mutants.

## Supplementary Material

Appendix 01 (PDF)

Dataset S01 (XLSX)

## Data Availability

Previously published data were used for this work (6). All other data are included in the manuscript and/or *SI Appendix*.
